# Source Analysis and Contamination Assessment of Potentially Toxic Element in Soil of Small Watershed in Mountainous Area of Southern Henan, China

**DOI:** 10.3390/ijerph192013324

**Published:** 2022-10-15

**Authors:** Hang Chen, Wei Wu, Li Cao, Xiaode Zhou, Rentai Guo, Liwei Nie, Wenxing Shang

**Affiliations:** 1 State Key Laboratory of Eco-hydraulics in Northwest Arid Region, Xi’an University of Technology, Xi’an 710048, China; 2 Binhai College, Nankai University, Tianjin 300000, China; 3 School of Water Resources and Environment, Chang’an University, Xi’an 710048, China

**Keywords:** potentially toxic element, matter element extension model, toxicity response coefficient, contamination assessment, PMF model

## Abstract

In this study, the concentrations of potentially toxic elements in 283 topsoil samples were determined. Håkanson toxicity response coefficient modified matter element extension model was introduced to evaluate the soil elements contamination, and the results were compared with the pollution index method. The sources and spatial distribution of soil elements were analyzed by the combination of the PMF model and IDW interpolation. The results are as follows, 1: The concentration distribution of potentially toxic elements is different in space. Higher concentrations were found in the vicinity of the mining area and farmland. 2: The weight of all elements has changed significantly. The evaluation result of the matter-element extension model shows that 68.55% of the topsoil in the study area is clean soil, and Hg is the main contamination element. The evaluation result is roughly the same as that of the pollution index method, indicating that the evaluation result of the matter-element extension model with modified is accurate and reasonable. 3: Potentially toxic elements mainly come from the mixed sources of atmospheric sedimentation and agricultural activities (22.59%), the mixed sources of agricultural activities and mining (20.26%), the mixed sources of traffic activities, nature and mining (36.30%), the mixed sources of pesticide use and soil parent material (20.85%).

## 1. Introduction

As an active part of the earth circle, the soil is not only the carrier of human survival but also a valuable resource of agricultural production. Therefore, soil contamination prevention and control have gradually become the focus of attention of scholars at home and abroad, among which Potentially toxic element contamination in soils is one of the most intractable problems in the process of soil ecological restoration [1,2,3]. The reason is that compared with other pollutants, potentially toxic elements in soils have concealment, irreversibility, long-term and latent. Once the content of elements exceeds the carrying capacity of the soil itself, it will directly endanger the yield and quality of crops, thus affecting human health and threatening the safety of human survival [4,5]. Many researchers have quantified the accumulation of potentially toxic elements in soils of typical regions around the world and found that soils in most regions are polluted by multiple elements [6,7,8,9]. According to the United States Environmental Protection Agency (US EPA), the contamination of potentially toxic elements in soil has caused physical problems for more than 10 million people worldwide [10].

The common methods for evaluating potentially toxic elements contamination in soil are single factor method, Nemerow index method, pollution load index method and ecological risk index method. Among them, single factor method and ecological risk index method do not consider contamination factors synthetically. Nemerow index method is difficult to highlight the qualitative change process of single factor, while the contamination load index method does not consider the difference of pollutant background value [11,12,13,14]. The matter-element matrix founded by Cai Wen takes the contamination index and characteristic value as matter-element, establishes the classical domain, node domain, and correlation degree through the measured data and evaluation criteria, and finally establishes the comprehensive evaluation system. The extension model is an organic combination of matter-element theory and extension set theory [15,16], which can objectively reflect the overall situation of the object to be evaluated. Some studies have shown that the matter-element extension model has higher accuracy and reliability than other evaluation methods [17,18,19,20,21,22]. However, there are few studies on the application of the matter-element extension model to the assessment of elements contamination in soil. 

At present, receptor models are widely used to analyze the source of potentially toxic elements in the soil. The receptor model is a kind of source analysis technique [23], which can qualitatively identify the contamination source types of soil samples and quantitatively determine the contribution rate of each contamination source. The receptor models mainly include principal component analysis, Unmix receptor model, isotopic labeling method, and PMF receptor model analysis [24,25,26]. Among them, the PMF method is widely used as a new method to analyze the source of potentially toxic elements in the soil. Previous research in the literature [27,28,29,30] analyzed the spatial distribution and sources of Pb, Cd, Cr, Hg, As, Cu, Zn, and Ni in cultivated land in Hangzhou, paddy fields in Shanghai, topsoil in Huairou District of Beijing and surrounding soil of Zhuxianzhuang Coal Mine in Anhui Province based on positive definite matrix factor method. It can be seen that the purpose of these studies is to analyze the sources of eight common potentially toxic elements in the soils of agriculture, urbanization, and mining areas. The research on contamination caused by Ba, Mo, Sb, and Ag in the soil at a watershed scale is very scarce. Although relevant studies have shown that human activities such as mining development, industrial pollutant emissions, chemical production, and pesticide use are the main sources of contamination caused by potentially toxic elements in soil [31,32,33,34,35,36,37], there are few reports on the sources of soil contamination factors in the mountainous areas of southern Henan. 

The Wangqiao River Basin is located in the mountainous area of the southern Henan Province is located in the northern foot of the Dabie Mountain. The climate and topographic conditions lead to serious soil and water loss. This makes it easier for potentially toxic elements to migrate, which will directly endanger the quality and yield of crops in this area, thus affecting human health and survival [38]. The small watershed in the mountainous area of Southern Henan as the research area, the matter-element extension model was used to evaluate potentially toxic elements contamination in the soil of the study area. Combined with GIS technology and PMF model to analysis the spatial distribution characteristics, sources and contribution rates of nine potentially toxic elements in soil of the study area, including Ag, Cu, Pb, Zn, Mo, Sb, Ba, As and Hg. This study is of great practical significance to strengthen the investigation and study of potential toxic elements in the soil of the small watershed in southern Henan, ensure the safety of people’s lives and property in this area, and build a good ecological environment in this area.

## 2. Materials and Methods

### 2.1. General Situation of the Study Area

**Figure 1:**
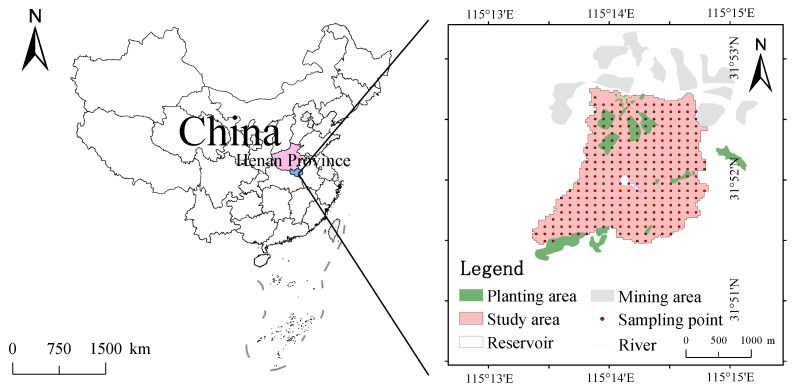
Overview of the study area and distribution of sampling sites.

The study area is located in Wangqiao Town, Shangcheng County, Xinyang City, Henan Province, with a geographical location of 115°11′30″~115°13′10″ E, 31°52′30″~31°53′30″ N (Figure 1). It is adjacent to Shuangpu town and Nianyushan Township in the East, Wuhe Township and Yuji town in the south, Bailu River in the west, across the river from Renhe Town, Huangchuan County, and shuangliushu Town, Huangchuan County in the north, belonging to the north foot of Dabie Mountain. The terrain is mainly low mountains and hilly areas. The domestic rivers belong to the Huaihe River basin, the main rivers are the Egret River, Vientiane River, Sidao River, and Qinyang River. The density of the river network is 0.5 km·km^−2^, and the largest river in the territory is the Egret River. The annual average temperature is 15.5 °C, the average temperature in January is 2 °C, and the annual rainfall is 1241.4 mm, which belongs to the subtropical monsoon climate. The main soil type is yellow-brown soil. The grain formation is fine sand (0.5~2.5 mm), silt (0.005~0.5 mm), coarse clay (0.01~0.005 mm), fine clay (0.001~0.01 mm) and micro-nanoparticles (<0.001 mm). The soil layer is deep and loose, and the pH value is 5.0~6.7. The content of organic matter is about 16~40 g·kg^−^^1^. The main types of land use are grassland, farmland, and forest land; the main vegetation types are evergreen broad-leaf forest, evergreen deciduous mixed forest, conifer broad-leaf mixed forest, and mountain meadow thicket. As the region is located between the Yangtze River and Huaihe River, at the junction of subtropical and warm temperate zones, with abundant rainfall and fertile land, it is mainly rich in tea, peanuts, sesame seeds, rape, paulownia, pears, peaches and so on. In recent years, the mining and smelting of metal minerals such as iron ore, silver mine, gold mine, and tin mine and non-metallic minerals such as clay loess, river sand, marble, granite, and shale have made contributions to the development of the regional economy [39]. However, a large number of metals waste residue, tailings sand and wastewater have been generated in the production process, resulting in serious damage to the mining area and the surrounding ecological environment.

### 2.2. Sample Collection and Test

In June 2021, the grid method and 3S technology were used to collect 0~20 cm soil on the surface of the study area. One sample was collected within 0.014 km^2^. A total of 283 sampling points were set. The distribution of sampling points is shown in Figure 1. Before sampling, the surface debris was removed by a wooden shovel, and the four apex and center points of 5 m × 5 m square were sampled respectively. During the sampling process, about 500 g of 0~20 cm soil samples were collected from each sampling point. After mixing, 500 g samples were taken as soil samples representing the sampling point, and then packed into self-sealing bags to record the geographical location, altitude, geomorphology and other environmental information of the sampling points. The samples were brought back to the laboratory and place it in a refrigerated room below 4 °C. Wait for natural air drying, then crush and grind, finally pass the 0.15 mm nylon sieve, to be tested the content of each potentially toxic element.

The contents of As, Sb, and Hg in soil were determined by BAFS-8520 atomic fluorescence spectrophotometer, and the contents of Cu, Zn, and Ba in soil were determined by PinAAcle 900T atomic absorption spectrophotometer. The Hg analysis and detection process was tested according to the inspection rules of GB/T:22105.2-2008. As and Sb are tested according to the inspection procedures in the literature [40,41]. The determination of Zn and Cu was based on the determination of Cu and Zn in soil by flame atomic absorption spectrophotometry [42], and then the contents of Pb, Ag, and Mo in the sample are determined by XSERIES2 inductively coupled plasma mass spectrometer. The detection limit of each analytical method is equal to or superior to the requirement of DZ/T0295-2016 [43]. 

### 2.3. Matter Element Extension Analysis Method

(1) Establish the matter-element matrix of the level of element contamination in soil

The matter-element matrix *E* is a three-dimensional matrix composed of the object to be evaluated *ϕ*, the contamination factor *K* and the value *C*, which is recorded as ***E*** *=* (*ϕ*, *K*, *C*). If the object *ϕ* has *n* contamination factors corresponding to *n* values, which is defined as follows:

(1) $$E=\left(\phi ,K_{n},C_{n}\right)=(\begin{array}{ccc} \phi & K_{1} & C_{1}\\ & K_{2} & C_{2}\\ & \vdots & \vdots \\ & K_{n} & C_{n} \end{array})$$

(2) Determining the classical domain and node domain of the element to be judged

The matter element matrix of the classical domain *E*(*j*) and node domain *E*(*p*) of the level of element contamination in soil is as follows:

(2) $$\begin{array}{c} E\left(j\right)=\left(\phi_{j},K_{i},C_{i}\right)=\left(\begin{array}{ccc} \phi & K_{1} & \left(\alpha_{j1,}\beta_{j1}\right)\\ & K_{2} & \left(\alpha_{j2,}\beta_{j2}\right)\\ & \vdots & \vdots \\ & K_{n} & \left(\alpha_{jn,}\beta_{jn}\right) \end{array}\right) \end{array}$$

(3) $$\begin{array}{c} E\left(\phi \right)=\left(\phi_{p},K_{i},C_{i}\right)=\left(\begin{array}{ccc} \phi_{p} & K_{1} & \left(\alpha_{p1,}\beta_{p1}\right)\\ & K_{2} & \left(\alpha_{p2,}\beta_{p2}\right)\\ & \vdots & \vdots \\ & K_{n} & \left(\alpha_{pn,}\beta_{pn}\right) \end{array}\right) \end{array}$$

where (*α_jn_*, *β_jn_*) is the range of grade *j* corresponding to the contamination factor *K_n_*; and (*α_pn_*, *β_pn_*) is the value range of matter element with respect to the contamination factor *K_n_*, obviously, (*α_jn_*, *β_jn_*) ⊂ (*α_pn_*, *β_pn_*) (*n* = 1, 2, 3, …, *i*).

(3) Determine the correlation degree function and calculate the correlation degree *T_j_*(*C_i_*)

In matter-element extension analysis, the correlation degree function indicates that the element to be judged meets the required value range, *T_j_*(*C_i_*) is the degree to which the contamination factor *X_i_* of the matter element to be judged conforms to the evaluation grade *j*. *T_j_*(*C_i_*) ≤ −1 indicates that the element to be evaluated does not meet the standard; −1 < *T_j_*(*C_i_*) ≤ 0 indicates that the element to be evaluated does not meet the standard, but has the condition that it can be transformed into a standard; *T_j_*(*C_i_*) ≥ 0 indicates that the element to be evaluated meets the standard [19]. The correlation degree function is:

(4) $$T_{j}\left(C_{i}\right)=\{\begin{array}{ll} -\frac{\lambda \left(C_{i},C_{ji}\right)}{\left|C_{ji}\right|} & C_{i}\in C_{ji}\\ \frac{\lambda \left(C_{i},C_{ji}\right)}{\lambda \left(C_{i},C_{pi}\right)-\lambda \left(C_{i},C_{ji}\right)} & C_{i}\notin C_{ji} \end{array}$$

(5) $$\begin{array}{c} C_{ji}=\left|\beta_{jn}-\alpha_{jn}\right| \end{array}$$

(6) $$\begin{array}{c} \lambda \left(C_{i},C_{ji}\right)=\left|C_{i}-\left(\beta_{jn}+\alpha_{jn}\right)\times 0.5\right|-\left(\beta_{jn}-\alpha_{jn}\right)\times 0.5 \end{array}$$

(7) $$\begin{array}{c} \lambda \left(C_{i},C_{pi}\right)=\left|C_{i}-\left(\beta_{pn}+\alpha_{pn}\right)\times 0.5\right|-\left(\beta_{pn}-\alpha_{pn}\right)\times 0.5 \end{array}$$

where *λ*(*C_i_*, *C_ji_*) and *λ*(*C_i_*, *C_pi_*) are the distance from the point *C_i_* to the classical domain *C_ji_* and node domain *C_pi_*.

(4) Calculate the comprehensive correlation degree

(8) $$\begin{array}{c} T_{j}\left(\phi \right)=\sum_{i=1}^{n}W_{ki}\times T_{j} \end{array}$$

where *T_j_*(*ϕ*) is the comprehensive correlation degree, *W_ki_* is the weight corresponding to *K_i_*, and max{*T_j_*(*ϕ*)} belongs to grade *j*, that is, contamination level of the element corresponding to the object *ϕ* to be evaluated.

The traditional weight coefficient is the multiple super-scale weighting method [13], which can not highlight the toxicity of low concentration and high toxicity element. Therefore, the toxicity response coefficient of Håkanson potentially toxic elements (Zn = 1, Ba = 2, Cu = Pb = 5, As = 10, Ag = Mo = 15 and Sb = Hg = 40) is introduced to modify the traditional metals evaluation weight coefficient [44,45], which can be calculated as follows:

The multiple super-scale weighting method:

(9) $$\begin{array}{c} W_{ki}=(C_{ki}/\bar{\Psi_{i}})/\left(\sum_{i=1}^{n}C_{ki}/\bar{\Psi_{i}}\right) \end{array}$$

Correction weight coefficient:

(10) $$\begin{array}{c} W_{ki}^{\prime }=(W_{ki}\times T_{r}^{i})/\left(\sum_{i=1}^{n}W_{ki}\times T_{r}^{i}\right) \end{array}$$

where $\bar{\Psi_{i}}$ is the arithmetic average of the evaluation grade, *W_ki_* is the conventional weight coefficient calculated by the multiple super-scale weighting method, *W_k_*′ is the modified weight coefficient, and *T*_r_*^i^* is the toxic response coefficient.

(5) Evaluation criteria

In this paper, the soil environmental background value of the main elements in Henan Province and GB15618-2018 [46], as well as the relevant reports at home and abroad, were used to determine the evaluation criteria of soil potentially toxic elements contamination in soil of the study area in Table 1 [17,18,19,20,21,22]. The evaluation criteria were divided into five grades, which were clean, relatively clean, light contamination, moderate contamination, and severe contamination. Level I takes the background value of the soil environment in the Henan Province as the upper limit value, level II takes 0.3 times the control standard as the upper limit value, level III takes the control standard 0.7 times as the upper limit value, level IV takes the control standard as the upper limit value, and level V takes the control standard 1.3 times as the upper limit value.

### 2.4. Pollution Index Method

Nemerow comprehensive pollution index (*P*_N_) [47] and Müller index (*I*_geo_) [48] are widely used in soil metal(s) pollution assessment, which can reflect the pollution grade and comprehensive pollution level of each factor. *P*_N_ and *I*_geo_ are used to evaluate soil elements pollution in the small watershed in the mountainous area of Henan Province, to test the rationality and reliability of the matter-element extension model results of modified weight coefficient.

#### 2.4.1. Nemerow Comprehensive Pollution Index (*P*_N_)

Nemerow comprehensive pollution index method (*P*_N_) is a method to comprehensively evaluate the pollution of many elements in soil. Which can be calculated as follows:

(11) $$\begin{array}{c} P_{N}=\sqrt{\frac{{\left(C_{i}/D_{i}\right)}_{\max}^{2}+{\left(C_{i}/D_{i}\right)}_{\mathrm{ave}}^{2}}{2}} \end{array}$$

where *P*_N_ is the comprehensive pollution index value of elements in the soil, *C_i_* is the measured concentration of element *i* in the soil, *D_i_* is the background value concentration of Xinyang City of element *i* in the soil [49]. According to the *P*_N_ value, the soil element pollution level can be divided into five grades, *P*_N_ ≤ 0.7 (Safety), 0.7 < *P*_N_ ≤ 1 (Warning value), 1 < *P*_N_ ≤ 2 (Mild pollution), 2 < *P*_N_ ≤ 3 (Moderate pollution), *P*_N_ > 3 (heavy pollution).

#### 2.4.2. Müller Index Method (*I*_geo_)

The Müller index method (*I*_geo_) not only fully considers the influence of the natural geological process on the background value of soil elements, but also pays attention to the influence of human activities on the environment. Which can be calculated as follows:

(12) $$\begin{array}{c} I_{\mathrm{geo}}=\log_{2}^{\left(C_{i}/K\times D_{i}\right)} \end{array}$$

where *I*_geo_ is the Müller index value, *C_i_* is the measured concentration of element *i* in the soil, *D_i_* is the background value concentration of Xinyang City of element *i* in the soil, *K* is the correction coefficient (the value of *K* is 1.5) [50]. The evaluation grades of Müller index method were as follows: *I*_geo_ < 0 (Non-pollution), 0 ≤ *I*_geo_ < 1 (Mild pollution), 1 ≤ *I*_geo_ < 2 (Mild and moderate pollution), 2 ≤ *I*_geo_ < 3 (Moderate pollution), 3 ≤ *I*_geo_ < 4 (Medium intensity pollution), 4 ≤ *I*_geo_ < 5 (Intensity pollution) and *I*_geo_ ≥ 5 (Super intensity pollution).

### 2.5. PMF Method

In this paper, the PMF 5.0 model developed by the United States Environmental Protection Agency (EPA) is used to analyze the source of potentially toxic elements in the soil of the small watershed in the mountainous area of southern Henan [51]. PMF is a source analysis technique based on the receptor model to qualitatively identify the contamination source types of soil samples and quantitatively determine the contribution rate of each contamination source. Paatero first proposed the PMF non-negative factor model in 1994 [52]. The difference between the PMF model and other methods is that the PMF model makes optimal use of the error analysis of the measured data points, and makes non-negative constraints on the decomposition of the factor matrix in the process of solving the problem, so the analysis results have more practical physical meaning [53]. The basic principle of source analysis of the PMF model is as follows: assuming that the sample concentration data matrix ***E****_ik_* can be decomposed into factor fraction matrix $A_{ij}$, factor load matrix $B_{jk}$ and residual matrix $\delta_{ik}$, the basic equations are as follows:

(13) $$\begin{array}{c} E_{ik}=\sum_{j=1}^{p}A_{ij}\times B_{jk}+\delta_{ik}i=1,2,\ldots ,m;k=1,2,\ldots ,n \end{array}$$

where ***E****_ik_* is the concentration of contamination factor *k* in sample point *i*; ***A****_ij_* is the contribution of sample point *i* in source *j*, that is, the fractional matrix; ***B****_jk_* is the contribution concentration of contamination factor *k* in source *j*, that is, the source load matrix; ***δ****_ik_* is the residual matrix.

The PMF model is mainly based on the weighted least square method to iteratively decompose the original matrix many times, obtain the optimal matrices ***A*** and ***B***, and thus obtain the minimum objective function *Q*:

(14) $$\begin{array}{c} \psi =\sum_{i=1}^{n}\sum_{k=1}^{m}{\left(\frac{S_{ik}-\sum_{j=1}^{p}A_{ij}\times B_{jk}}{\mu_{ik}}\right)}^{2}=\sum_{i=1}^{n}\sum_{k=1}^{m}{\left(\frac{\delta_{ik}}{\mu_{ik}}\right)}^{2} \end{array}$$

where *Q* is the cumulative residual, *μ_ik_* is the uncertainty of the concentration of contamination factor *k* in sample point *i*, which can be calculated as follows:

(15) $$\begin{array}{c} \mu_{ik}=0.1S_{ik}+MDL/3 \end{array}$$

where *MDL* is the detection limit of each substance, ***S****_ik_* is the concentration of potential toxic element in soil samples.

### 2.6. Data Processing

The statistical analysis and contamination evaluation of the content of potentially toxic elements in the surface soil of the study area was carried out in IBM SPSS Statistics 26, Excel 365, and Origin pro 2021 software. EPA PMF 5.0 software was used to analyze the source of potentially toxic elements in soil, and then ArcGIS 10.4 software was used to interpolate potentially toxic elements content in soil to obtain spatial distribution. Finally, Adobe illustrator 2020 was used to process the image.

## 3. Results and Discussions

### 3.1. Statistical Characteristics of Potentially Toxic Elements Content in Soil

The average values of potentially toxic elements *ω*(Ag), *ω*(Cu), *ω*(Pb), *ω*(Zn), *ω*(Mo), *ω*(S), *ω*(Ba), *ω*(As), and *ω*(Hg) in the topsoil of the study area was 0.066, 30.5, 25.09, 73.52, 1.057, 1.056, 530.9, 13.16 and 0.058 mg·kg^−^^1^ in Table 2. Among them, except Ag and Mo, the average values of other elements were higher than the soil background values of Henan Province to varying degrees. The ratio with the background value of soil environment in Henan Province was 0.94, 1.39, 1.06, 1.2, 0.67, 1.17, 1.06, 1.32, and 1.93, respectively. The proportion of sample points in excess of background value was 34.63%, 61.13%, 52.3%, 63.25%, 10.95%, 53.36%, 52.3%, 56.54% and 75.97%. Compared with the screening value of soil pollution risk in agricultural land, the proportion of soil Cu, Zn, and As exceeding the standard was 2.21%, 4.63%, and 3.53%. This indicated that might have ecological risks and might be harmful to crops in the areas. The results show that if the coefficient of variation of element is more than 0.5, the spatial distribution of the element content is uneven and there is a risk of point source contamination, which may be caused by the entry of foreign substances [54,55]. Therefore, the greater the coefficient of variation, the more serious the soil interference. The coefficient of variation of elements in soil was Cu(0.835) > As(0.686) > Hg(0.669) > Mo(0.487) > Sb(0.49) > Ag(0.452) > Zn(0.435) > Ba(0.349) > Pb(0.289). The coefficients of variation of Cu, As and Hg were all greater than 0.5, which may be affected by human activities such as planting, farming, and mine excavation. 

**Figure 2:**
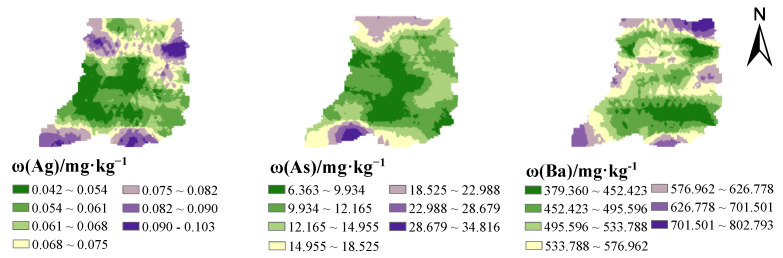

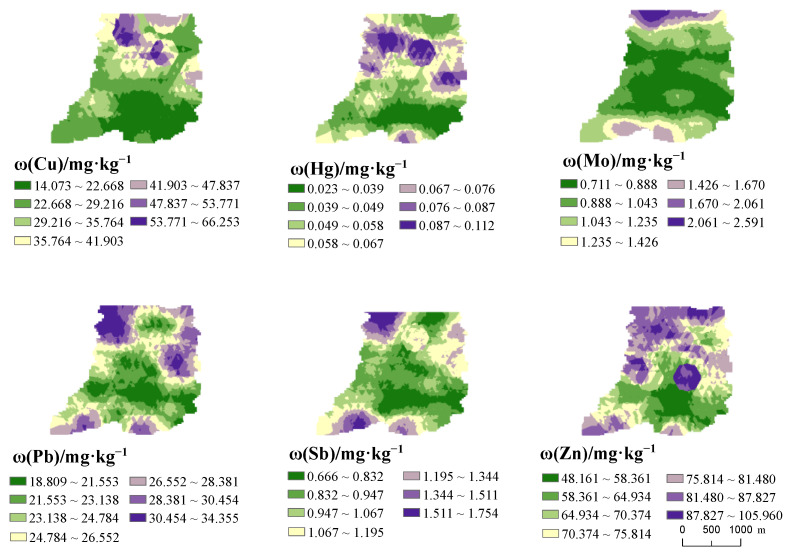
Spatial distribution of potentially toxic elements content in soil of the study area.

The spatial distribution of potentially toxic elements in the topsoil of the study area is shown in Figure 2. Nine elements in the soil of the study area are distributed in an island and sheet shape, among which the high-value distribution of Ba and Cu is scattered. The other high-value areas of elements are mainly concentrated in the north and south of the study area, and the contents of elements in the central region are relatively low. Combined with the analysis from Figure 1, the contents of Cu, Ag, and Ba near the mining area were shown to be significantly higher. The results show that the mining of the mine has a certain effect on the content of metals in the topsoil of this area.

### 3.2. Potentially Toxic Elements Contamination Assessment in Topsoil

#### 3.2.1. Evaluation of Element Contamination by Matter-Element Extension Analysis

In this experiment, a total of 283 surface soil samples in the study area were collected, and the first sample A1 was selected as an example to calculate. The contents of potentially toxic elements (Ag, Cu, Pb, Zn, Mo, Sb, Ba, As, and Hg) in sample A1 were 0.09, 36.29, 37.38, 124.9, 1.358, 1.624, 526, 19.193 and 0.095 mg·kg^−1^.

(1) It is determined that the matrix of the matter element of ***E***_1_ is:

$$E_{1}=\left(\begin{array}{ccc} \phi_{1} & \mathrm{Ag} & 0.09\\ & \mathrm{Cu} & 36.29\\ & \begin{array}{c} \mathrm{Pb}\\ \mathrm{Zn}\\ \begin{array}{c} \mathrm{Mo}\\ \mathrm{Sb}\\ \begin{array}{c} \mathrm{Ba}\\ \mathrm{As}\\ \mathrm{Hg} \end{array} \end{array} \end{array} & \begin{array}{c} 37.38\\ 124.9\\ \begin{array}{c} 1358\\ 1.624\\ \begin{array}{c} 526\\ 19.193\\ 0.095 \end{array} \end{array} \end{array} \end{array}\right)E\left(3\right)=\left(\begin{array}{ccc} N_{3} & \mathrm{Ag} & \left(87.6,\;204.4\right)\\ & \mathrm{Cu} & \left(120,\;280\right)\\ & \begin{array}{c} \mathrm{Pb}\\ \mathrm{Zn}\\ \begin{array}{c} \mathrm{Mo}\\ \mathrm{Sb}\\ \begin{array}{c} \mathrm{Ba}\\ \mathrm{As}\\ \mathrm{Hg} \end{array} \end{array} \end{array} & \begin{array}{c} \left(150,\;350\right)\\ \left(150,\;350\right)\\ \begin{array}{c} \left(139.5,\;325.5\right)\\ \left(12,\;28\right)\\ \begin{array}{c} \left(1668,\;3892\right)\\ \left(12,\;28\right)\\ \left(0.45,\;1.05\right) \end{array} \end{array} \end{array} \end{array}\right)$$

(2) According to Table 1, establishment of classical domain matrices ***E***(1), ***E***(2), ***E***(3), ***E***(4), ***E***(5) and node field matrices ***E***(*p*):

$$\begin{array}{cc} E\left(1\right)=\left(\begin{array}{ccc} N_{1} & \mathrm{Ag} & \left(0,\;0.07\right)\\ & \mathrm{Cu} & \left(0,\;22\right)\\ & \begin{array}{c} \mathrm{Pb}\\ \mathrm{Zn}\\ \begin{array}{c} \mathrm{Mo}\\ \mathrm{Sb}\\ \begin{array}{c} \mathrm{Ba}\\ \mathrm{As}\\ \mathrm{Hg} \end{array} \end{array} \end{array} & \begin{array}{c} \left(0,\;23.6\right)\\ \left(0,\;61.5\right)\\ \begin{array}{c} \left(0,\;0.57\right)\\ \left(0,\;0.9\right)\\ \begin{array}{c} \left(0,\;502\right)\\ \left(0,\;10\right)\\ \left(0,\;0.025\right) \end{array} \end{array} \end{array} \end{array}\right) & E\left(2\right)=\left(\begin{array}{ccc} N_{2} & \mathrm{Ag} & \left(0.07,\;87.6\right)\\ & \mathrm{Cu} & \left(22,\;120\right)\\ & \begin{array}{c} \mathrm{Pb}\\ \mathrm{Zn}\\ \begin{array}{c} \mathrm{Mo}\\ \mathrm{Sb}\\ \begin{array}{c} \mathrm{Ba}\\ \mathrm{As}\\ \mathrm{Hg} \end{array} \end{array} \end{array} & \begin{array}{c} \left(23.6,\;150\right)\\ \left(61.5,\;150\right)\\ \begin{array}{c} \left(0.57,\;139.5\right)\\ \left(0.9,\;12\right)\\ \begin{array}{c} \left(502,\;1668\right)\\ \left(10,\;12\right)\\ \left(0.025,\;0.45\right) \end{array} \end{array} \end{array} \end{array}\right)\\ E\left(3\right)=\left(\begin{array}{ccc} N_{3} & \mathrm{Ag} & \left(87.6,\;204.4\right)\\ & \mathrm{Cu} & \left(120,\;280\right)\\ & \begin{array}{c} \mathrm{Pb}\\ \mathrm{Zn}\\ \begin{array}{c} \mathrm{Mo}\\ \mathrm{Sb}\\ \begin{array}{c} \mathrm{Ba}\\ \mathrm{As}\\ \mathrm{Hg} \end{array} \end{array} \end{array} & \begin{array}{c} \left(150,\;350\right)\\ \left(150,\;350\right)\\ \begin{array}{c} \left(139.5,\;325.5\right)\\ \left(12,\;28\right)\\ \begin{array}{c} \left(1668,\;3892\right)\\ \left(12,\;28\right)\\ \left(0.45,\;1.05\right) \end{array} \end{array} \end{array} \end{array}\right) & E\left(4\right)=\left(\begin{array}{ccc} N_{4} & \mathrm{Ag} & \left(204.4,\;292\right)\\ & \mathrm{Cu} & \left(280,\;400\right)\\ & \begin{array}{c} \mathrm{Pb}\\ \mathrm{Zn}\\ \begin{array}{c} \mathrm{Mo}\\ \mathrm{Sb}\\ \begin{array}{c} \mathrm{Ba}\\ \mathrm{As}\\ \mathrm{Hg} \end{array} \end{array} \end{array} & \begin{array}{c} \left(350,\;500\right)\\ \left(350,\;500\right)\\ \begin{array}{c} \left(325.5,\;465\right)\\ \left(28,\;40\right)\\ \begin{array}{c} \left(3892,\;5560\right)\\ \left(28,\;40\right)\\ \left(1.05,\;1.5\right) \end{array} \end{array} \end{array} \end{array}\right)\\ E\left(5\right)=\left(\begin{array}{ccc} N_{5} & \mathrm{Ag} & \left(292,\;379.6\right)\\ & \mathrm{Cu} & \left(400,\;520\right)\\ & \begin{array}{c} \mathrm{Pb}\\ \mathrm{Zn}\\ \begin{array}{c} \mathrm{Mo}\\ \mathrm{Sb}\\ \begin{array}{c} \mathrm{Ba}\\ \mathrm{As}\\ \mathrm{Hg} \end{array} \end{array} \end{array} & \begin{array}{c} \left(500,\;650\right)\\ \left(500,\;650\right)\\ \begin{array}{c} \left(465,\;604.5\right)\\ \left(40,\;52\right)\\ \begin{array}{c} \left(5560,\;7728\right)\\ \left(40,\;52\right)\\ \left(1.5,\;1.95\right) \end{array} \end{array} \end{array} \end{array}\right) & E\left(p\right)=\left(\begin{array}{ccc} N_{p} & \mathrm{Ag} & \left(0,\;379.6\right)\\ & \mathrm{Cu} & \left(0,\;520\right)\\ & \begin{array}{c} \mathrm{Pb}\\ \mathrm{Zn}\\ \begin{array}{c} \mathrm{Mo}\\ \mathrm{Sb}\\ \begin{array}{c} \mathrm{Ba}\\ \mathrm{As}\\ \mathrm{Hg} \end{array} \end{array} \end{array} & \begin{array}{c} \left(0,\;650\right)\\ \left(0,\;650\right)\\ \begin{array}{c} \left(0,\;604.5\right)\\ \left(0,\;52\right)\\ \begin{array}{c} \left(0,\;7728\right)\\ \left(0,\;52\right)\\ \left(0,\;1.95\right) \end{array} \end{array} \end{array} \end{array}\right) \end{array}$$

(3) potentially toxic elements weight correction

**Figure 3:**
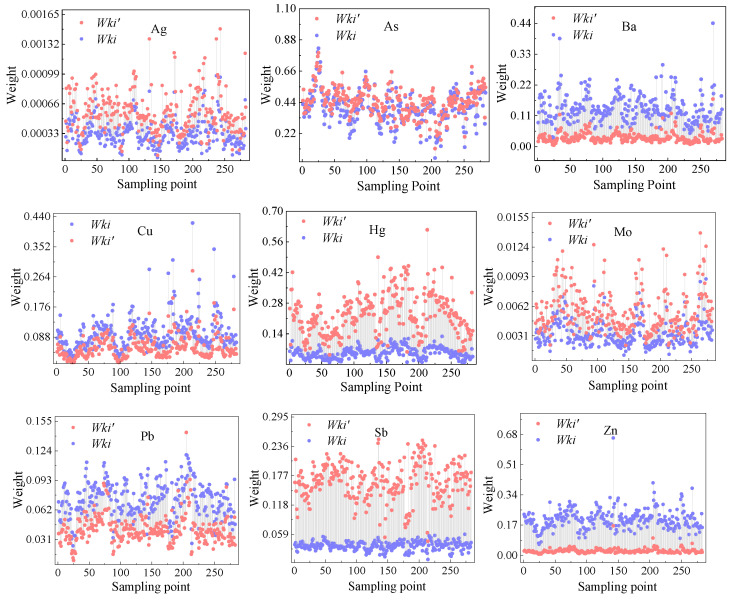
Comparative Analysis of weight values of potentially toxic elements in soil of the study area.

The weight values of nine elements in the study area calculated by the traditional multiple super-scale weighting method were compared with the weight values corrected by introducing the toxicity response coefficient (Figure 3). It can be seen that the weight values of all elements have changed significantly. Except that the modified weight *W_ki_*′ of Ba, Cu, Pb, and Zn is smaller than the weight value *W_ki_* of the traditional multiple super-scale weighting method, the decrease is 41.07%, 41.89%, 80.03%, and 75.98%. The modified weight values of the other five elements increased in varying degrees, with the increase as follows: As < Ag < Mo < Hg < Sb, in which the As increased the least, around 11.33%, Sb increased the most, by approximately 352.84%. This is due to the introduction of the Håkanson toxicity response coefficient to modify the traditional over-standard multiple weighting method, and the *W_k_*′ value is proportional to the introduced toxic response coefficient. The greater the toxicity of the element, the greater the increase of the element’s weight. Therefore, *W_ki_*′ not only reflects the toxicity of elements, but can also emphasize the difference in the cumulative concentration of potentially toxic elements, compared with the traditional weight assignment method is more practical significance.

(4) Evaluation results

According to Formulas (4)–(7), the single index correlation *K_j_*(*φ_i_*) of 283 matter elements to be judged with respect to each evaluation grade is calculated. The higher the *K_j_*(*φ_i_*) value is, the higher the belonging degree of the contamination factor to the evaluation grade is. For the same matter-element, the correlation degree of different contamination factors is different, so the contamination grade is also different, showing the incompatibility between each sample point. According to Formula (8) and the above correction weight, the comprehensive correlation degree of each matter element was calculated and the grade evaluated.

**Figure 4:**
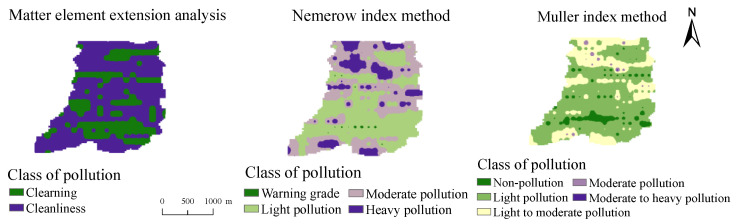
Spatial distribution of assessment grades of potential toxic elements contamination in topsoil in the study area.

Taking sample A1 as an example, the correlation degree of comprehensive indexes was −0.339, 0.901, −0.194, −0.655 and −0.758, in which the maximum comprehensive correlation degree max{*T*_2_(*N*)} = 0.901. It can be determined that the sample A1 belongs to grade II and is in a cleanliness state. According to the above principles, the levels of potential toxic elements contamination of 283 topsoil samples from small watershed in mountainous area of Southern Henan were calculated. The distribution of the evaluation results of matter-element extension analysis method about potential toxic element contamination in soil of the study area was obtained by using Spatial Analyst tool in ArcGIS 10.4 and IDW interpolation method (Figure 4). The results of matter-element extension analysis showed that 31.45% of the topsoil was grade I clean soil, and the rest of the soil was grade II cleanliness soil. On the whole, the contamination level of elements in soils of the study area is mainly clean, and Hg was the main contamination element.

#### 3.2.2. Evaluation Results of Nemerow Comprehensive pollution Index method

Formula (11) was used to evaluate the potentially toxic elements in the topsoil of the study area, and the spatial distribution of the evaluation results of the Nemerow comprehensive pollution index method in the study area was analyzed and studied by using the ArcGIS software platform (Figure 4). The results showed that the variation range of Nemerow comprehensive pollution index (*P*_N_) in the study area was 0.87 ≤ 9.94, with an average of 2.24. The contamination evaluation grade of 14 sampling points in the study area was grade II (warning line), accounting for 4.95% of the total, and the evaluation grade of 137 samples was grade III (mild pollution), accounting for 48.41% of the total, of which Hg contributed the most, and 132 samples were moderately and severely polluted, accounting for 46.64% of the total. Most of these polluted areas are distributed in the central and northern parts of the study area, indicating that the seriously polluted areas are affected by mining to a certain extent.

#### 3.2.3. Evaluation Results of Müller Index Method

By using Formula (12), the ground accumulation index of the potentially toxic elements in the surface soil of the study area was calculated. The average value of the ground cumulative index of nine potentially toxic elements is Ag < Ba < Pb < Sb < As < Zn < Cu < Mo < Hg, in which the average value of Ag was −0.8 and the average value of Hg was 0.327. Considering the influence of a high concentration of potentially toxic elements at specific points on the environment, the maximum value was selected from the Müller index of nine elements, and the level of element contamination at the target point was evaluated by this value, then the spatial distribution of element contamination assessment in the study area based on the Müller index method was drawn by ArcGIS software (Figure 4). Figure 4 shows that 18.73% of the areas in the study area belong to grade I pollution-free. 46.29% of the areas belong to II mild pollution area, 34.98% of the areas reached grade III and above a mild to moderate intensity contamination. The spatial distribution of contamination area is about the same as that of Nemerow index method. Most of the polluted areas are concentrated in the central and northern parts of the study area, and the contamination grade of potential toxic element in the southern region is relatively low.

### 3.3. Correlation Analysis of Potential Toxic Elements in Soil

**Figure 5:**
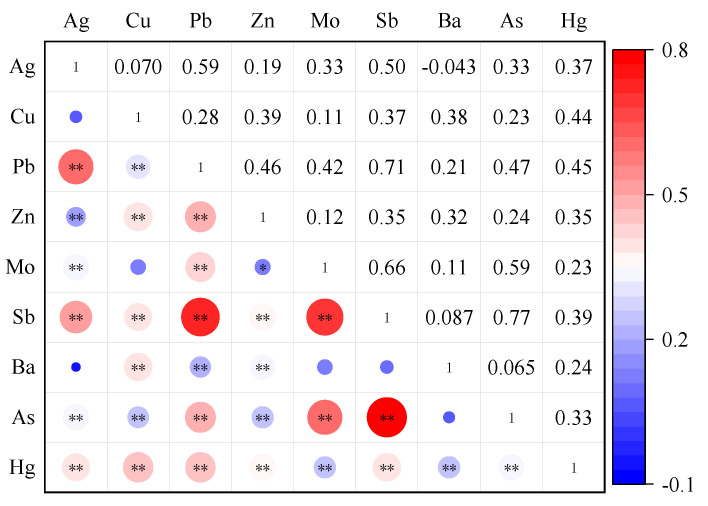
Correlation coefficient of potential toxic elements in soil. Note: * means *p* < 0.05, ** means *p* < 0.01.

The correlation analysis results of potentially toxic elements in the topsoil of the study area are shown in Figure 5. Some studies have shown that the greater the correlation coefficient between elements in soil, the more likely it is to come from the same source, or there may be multiple contamination sources [56]. Figure 5 shows that the correlation coefficients among Pb-Ag, Pb-Sb and Mo-Sb-As are all greater than 0.5. The significant test of 0.01 level shows that these elements have the same contamination source and may have compound contamination. The correlation between Ba and other elements was not significant, indicating that it has different sources from other elements.

### 3.4. Analysis on the Source of Potential Toxic Elements in Soil

The experimental data were imported into EPA PMF 5.0 software (Developed by Environmental Protection Agency, Washington, D.C, USA), and the signal-to-noise ratio (SNR) of contamination factors was more than 2.3, which was classified as a “strong” variable per the requirements of model calculation. After the “Robust” mode was set up, the number of runs, factors, and seeds, ran the model. Setting different factor number scenarios has a significant impact on the source analysis results of the model. In this study, 3~6 factor scenarios were set up to calculate the PMF source analysis results under each factor scenario. The results have shown that the number of factors was optimal when Q_robust_/Q_expected_ drops rapidly [57]. In this experiment, when the number of factors changed from 3 to 4, Q_robus_/Q_exected_ decreased greatly from 3.52 to 2.63. Therefore, the optimal number of factors for this study was determined to be 4. At this time, the measured content of elements and the predicted values of the model reached the best fitting effect, and the residual difference of most substances was between −3~3, the results are given in Table 3. Except for the fitting curve *R*^2^ of Zn is 0.397, the fitting curve *R*^2^ of other elements is greater than 0.5, which indicates that the source analysis results of the PMF model are better as a whole, and the model is stable under the scenario of 4 factors.

**Figure 6:**
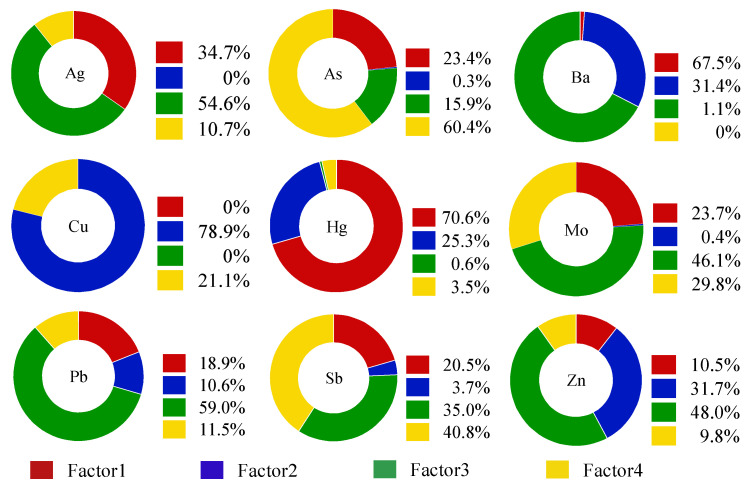
Contribution rate of each factor to potential toxic element concentration distribution.

The PMF model source analysis results are shown in Figure 6. It can be seen from the diagram that the main load element of factor 1 is Hg, and the contribution rate is 70.57%. It can be seen from Table 2 that the coefficient of variation of Hg is more than 0.6, which belongs to strong variability, indicating that its source is mainly affected by human activities. Previous studies have shown that Hg in farmland mainly comes from non-point source pollution, and its main sources are atmospheric deposition and the use of chemical fertilizers in agriculture [58,59,60,61]. Through field investigation, it was found that some farmers in the study area used coal as their main fuel, and that the source of heating duringwinter in the north was also mainly coal. The discharged Hg enter the atmosphere in the form of steam and then transfer to the soil through atmospheric deposition, resulting in the increase of Hg content in the soil. Therefore, factor 1 is the mixed sources of atmospheric sedimentation and agricultural activities.

In factor 2, Cu occupies a large load, and its contribution rate is 78.9%. Figure 1 and Figure 2 show that the areas with high Cu content are mainly distributed near the mining and planting area, and the point concentration near the mining area is higher than that of the farmland. Studies have shown that a large amount of CuSO_4_ is used as an insecticide for fruit trees in pesticide production [62,63]. Through the field survey, it can be seen that pears, peaches, apricots and other fruit trees are planted in the study area. Local farmers use pesticides such as suspended copper and copper to increase yield and avoid pests, which may lead to the accumulation of Cu in the topsoil of the planting area. In addition, it can be seen from Table 2 that the coefficient of variation of Cu is large, which indicates that there may be point source pollution, and in the mining process, a large number of Cu tailings and residues will accumulate on the surface and spread to the surrounding area under the leaching of Rain Water, thus moving to the soil, resulting in the increase of Cu content in the surrounding soil. Therefore, factor 2 can be determined as the mixed sources of agricultural activities and mining.

Factor 3 contributed to Ba, Pb, Ag, Zn, Mo and Sb, and the contribution rates were 67.5%, 59%, 54.55%, 48%, 46.4% and 35%. Ba is an alkaline earth metal, located in the sixth cycle of the periodic table IIA family, and is the most active alkaline earth metal element. Some studies have shown that the main sources of Ba in natural soil are natural parent material and mining [64,65]. However, the average value of Ba content in soils in the study area is 530.9 mg·kg^−1^, which is much higher than the soil environmental background value of Henan Province (502 mg·kg^−1^), so the possibility of coming from natural parent material is low. From the distribution of Ba elements in Figure 1 and Figure 2, it can be seen that the high-value area of Ba content is mainly concentrated in the middle and north of the study area, and is located near the mining area. Some studies have shown that the Mo element in soil mainly comes from Mo mineral, and after weathering, it enters the solution in the form of molybdate ion under the leaching of Rain Water, and thus migrates to the surface soil [66]. It can be seen from Table 2 that the average value of *ω*(Mo) in the study area is 1.057 mg·kg^−1^ which is lower than the background value of the soil environment in Henan Province, so Mo mainly comes from the natural parent material. The content of Ag in natural soil is very low. From the perspective of spatial distribution of Ag, the content of Ag in some sampling points in the study area is high, all of which are located near the mining area, mainly from tailings and waste residue in the process of mining development. Studies have confirmed that the main sources of Pb and Zn are motor vehicle exhausts, tire wear and gasoline additives [32,67,68]. Although unleaded gasoline has been widely used in Henan Province, there are still residues in the soil due to the fact that potential toxic elements in the soil are not easy to migrate. After the site survey, it was found that although the study area is far from the urban area, there is a traffic trunk line in the study area, these traffic pollutants increase the content of Pb and Zn in the topsoil through atmospheric deposition. Combined with Figure 1 and Figure 2, it shows that the high value area of Pb and Zn content is mainly located near the highway, so factor 3 can be judged as the mixed sources of traffic activities, nature and mining.

The elements with a large contribution rate in factor 4 were As and Sb, the contribution rates were 60.4% and 40.8%, and the correlation coefficients between them were 0.77, indicating that the two elements were more likely to have the same contamination source. Some studies have shown that the sources of As in soil are mainly man-made and natural sources. Generally, some arsenic-bearing sulfide and oxide rocks migrate to soil under weathering and rain erosion [69,70]. From Table 2, the average value of *ω*(As) in the study area is 13.16 mg·kg^−1^, it is obviously higher than the background value of soil environment in Henan Province (10 mg·kg^−1^). Although the use of pesticides containing As has been prohibited (such as fungicides, pesticides, and herbicides [71]), it can be seen from Figure 1 and Figure 2 that the high-value areas of As content are mainly distributed near the planting areas, and there are some areas where As pesticides are still used for a long time, resulting in the accumulation of As in farmland soils. Sb in soil mainly comes from the weathering of antimony-bearing rocks and the deposition of antimony dust in the atmosphere. Sb elements are relatively stable and have poor migration. Table 2 shows that the coefficient of variation of Sb elements is small, indicating that it is less subject to human interference, and the average value (1.056 mg·kg^−1^) of Sb is close to the background value of Henan Province (0.9 mg·kg^−1^), which further indicates that the main source of Sb is natural parent material. Therefore, factor 4 can be determined as the mixed sources of pesticide use and soil parent material.

**Figure 7:**
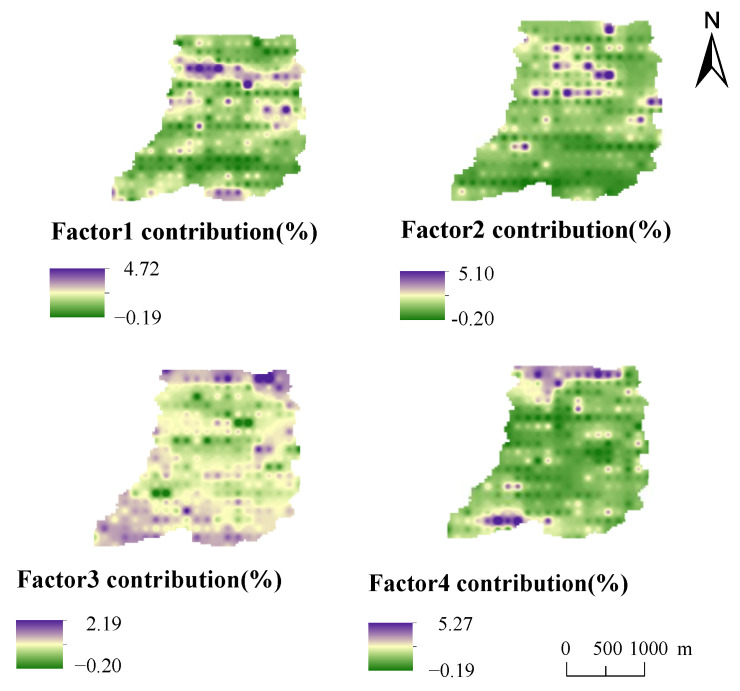
Spatial distribution of PMF source contribution.

To summarise, potential toxic element pollution in the soil of the study area is mainly the mixed source of atmospheric sedimentation and agricultural activities, the mixed source of agricultural activities and mining, the mixed source of traffic activities, nature and mining, the mixed source of pesticide use and soil parent material, accounting for 22.59%, 20.26%, 36.30%, and 20.85%. The IDW interpolation of the PMF model source analysis results were carried out by ArcGIS 10.4 (Developed by Environmental Systems Research Institute Inc, Redlands, CA, USA) to obtain the spatial distribution of different pollution sources in the study area, and the results are shown in Figure 7. It can be seen that the high-value areas contributed to by factor 3 and factor 4 are mainly distributed in the north and south of the study area, mainly occurring in a banded distribution, while the high-value regional distribution of factor 1 and factor 2 is discrete, mainly affected by the physical properties of pollutants, showing island distribution.

## 4. Conclusions

This study explored the spatial distribution of potentially toxic elements in the surface soil of a small watershed in southern Henan, assessed the contamination level of potentially toxic elements, analyzed the main sources of each element, and quantified the contribution rate of each contamination source. The results of a spatial analysis showed that nine elements in the soil of the study area are mainly island and banded, and the areas with high element content are mainly concentrated near the mining area and planting area. The weights of the matter-element extension model are modified by using the Håkanson toxicity response coefficient. The results show that the weights of all elements have changed significantly, with As increasing by 11.33%, the smallest, and Sb by 352.84%, the largest. The evaluation results of the matter-element extension model show that 31.45% of the topsoil in the study area is class I clean soil, the rest is class II cleanliness soil, and Hg is the main contamination element. The matter-element extension analysis method takes into account the content and toxicity of elements, and its evaluation results are more accurate and reasonable. Source quantification identified that transportation, nature, and mine development are the largest contributors to potential toxic element contamination (Ba, Pb, Ag, Zn, Mo, and Sb) in the soil of the study area, with a contribution rate of 36.30%. Atmospheric sedimentation and agricultural activities are the second largest contributors to the elements Hg, accounting for 22.59%. Pesticide use and soil parent material are the third largest factors contributing to As and Sb, accounting for 20.85%, Agricultural activities and mining are the fourth major factor of element contamination (Cu), with a contribution rate of 20.26%. This study not only provides a practical case for regional potential toxic element source analysis and contamination assessment using receptor model technology and assessment methods of soil potential toxic element contamination, but also provides a basis for the prevention and control of potential toxic element pollution in the study area.

## Figures and Tables

**Table 1:** Evaluation standard of potentially toxic elements contamination/mg·kg^−1^.

Contamination Factor	*ω*								
	Ag	Cu	Pb	Zn	Mo	Sb	Ba	As	Hg
Cleaning I	0.07	22	23.6	61.5	0.57	0.9	502	10	0.025
Cleanliness II	87.6	120	150	150	139.5	12	1668	12	0.45
Mild pollution III	204.4	280	350	350	325.5	28	3892	28	1.05
Moderate pollution IV	292	400	500	500	465	40	5560	40	1.5
heavy pollution V	379.6	520	650	650	604.5	52	7728	52	1.95

**Table 2:** Statistics of potentially toxic elements content in topsoil of the study area(*n* = 283).

Project	Ag	Cu	Pb	Zn	Mo	Sb	Ba	As	Hg
Background values/mg·kg^−1^	0.07	22	23.6	61.5	1.57	0.9	502	10	0.03
Limit of Detection/mg·kg^−1^	0.03	0.7	1	5	0.1	0.3	1	0.2	0.002
Average/mg·kg^−1^	0.066	30.5	25.09	73.52	1.057	1.056	530.9	13.16	0.058
Standard deviations/mg·kg^−1^	0.03	25.46	7.258	31.95	0.515	0.485	185	9.029	0.039
Maximum/mg·kg^−1^	0.177	208.5	51.17	404.8	5.998	3.553	1667	81.847	0.344
Minimum/mg·kg^−1^	0.021	7.222	11.24	31.65	0.459	0.336	193.4	0.75627	0.006
Coefficient of variation	0.452	0.835	0.289	0.435	0.487	0.459	0.349	0.686	0.669

**Table 3:** Fitting results of measured values and simulated predicted values of soil element content.

Element	*R* ^2^	Slope	Intercept
Ag	0.62193	0.579	0.0242
Cu	0.84915	0.6573	9.02897
Pb	0.899421	0.90508	2.05252
Zn	0.39664	0.40038	39.72587
Mo	0.53154	0.48908	0.44928
Sb	0.83869	0.79493	0.19022
Ba	0.97855	0.95017	24.12398
As	0.94961	0.87377	1.44268
Hg	0.9978	0.97296	0.00139
